# Supplementary material for: Individual and healthcare supply-related HIV transmission factors in HIV-positive patients enrolled in the antiretroviral treatment access program in the Centre and Littoral regions in Cameroon (ANRS-12288 EVOLCam survey)
Source: PLoS One. 2022 Apr 6;17(4):e0266451. doi: 10.1371/journal.pone.0266451 (PMC8985982; doi:10.1371/journal.pone.0266451)
Supplement: S1 Table — Abbreviations: ARV = Antiretroviral drugs HSP: HIV-service profile. (DOCX) [file pone.0266451.s001.docx]

**S1 Table. Characteristics of the 19 participating healthcare services and HIV service profiles (EVOLCam survey, ANRS-12288).**

|  | **Total  n=19** |  | **HSP 1** |  | **HSP 2** |  | **HSP 3** |  | **HSP 4** |
| --- | --- | --- | --- | --- | --- | --- | --- | --- | --- |
|  |  |  | **n=4** |  | **n=5** |  | **n=6** |  | **n=4** |
|  | **n (%)** |  | **n (%)** |  | **n (%)** |  | **n (%)** |  | **n (%)** |
| ***Service general characteristics*** |  |  |  |  |  |  |  |  |  |
| Location (region) |  |  |  |  |  |  |  |  |  |
| Centre | 11 (58) |  | 0 (0) |  | 3 (60) |  | 4 (67) |  | 4 (100) |
| Littoral | 8 (42) |  | 4 (100) |  | 2 (40) |  | 2 (33) |  | 0 (0) |
| Date of opening |  |  |  |  |  |  |  |  |  |
| ≤2001 | 7 (37) |  | 1 (25) |  | 5 (100) |  | 1 (17) |  | 0 (0) |
| >2001 | 12 (63) |  | 3 (75) |  | 0 (0) |  | 5 (83) |  | 4 (100) |
| Legal status |  |  |  |  |  |  |  |  |  |
| Public | 13 (68) |  | 3 (75) |  | 4 (80) |  | 4 (67) |  | 2 (50) |
| Private | 6 (32) |  | 1 (25) |  | 1 (20) |  | 2 (33) |  | 2 (50) |
| Type of HIV service |  |  |  |  |  |  |  |  |  |
| HIV Management Unit | 11 (58) |  | 4 (100) |  | 0 (0) |  | 3 (50) |  | 4 (100) |
| Accredited Treatment Center | 8 (42) |  | 0 (0) |  | 5 (100) |  | 3 (50) |  | 0 (0) |
| Number of beds |  |  |  |  |  |  |  |  |  |
| ≤100 | 12 (63) |  | 4 (100) |  | 0 (0) |  | 4 (67) |  | 4 (100) |
| >100 | 7 (37) |  | 0 (0) |  | 5 (100) |  | 2 (33) |  | 0 (0) |
|  |  |  |  |  |  |  |  |  |  |
| ***Number of healthcare providers*** |  |  |  |  |  |  |  |  |  |
| Number of physicians |  |  |  |  |  |  |  |  |  |
| ≤4 | 12 (63) |  | 2 (50) |  | 1 (20) |  | 5 (83) |  | 4 (100) |
| >4 | 7 (37) |  | 2 (50) |  | 4 (80) |  | 1 (17) |  | 0 (0) |
| Number of nurses |  |  |  |  |  |  |  |  |  |
| ≤2 | 11 (58) |  | 0 (0) |  | 2 (40) |  | 5 (83) |  | 4 (100) |
| >2 | 8 (42) |  | 4 (100) |  | 3 (60) |  | 1 (17) |  | 0 (0) |
| Number of nursing assistants |  |  |  |  |  |  |  |  |  |
| <1 | 11 (58) |  | 1 (25) |  | 3 (60) |  | 6 (100) |  | 1 (25) |
| ≥1 | 8 (42) |  | 3 (75) |  | 2 (40) |  | 0 (0) |  | 3 (75) |
| Number of psychologists |  |  |  |  |  |  |  |  |  |
| <1 | 12 (63) |  | 3 (75) |  | 2 (40) |  | 3 (50) |  | 4 (100) |
| ≥1 | 7 (37) |  | 1 (25) |  | 3 (60) |  | 3 (50) |  | 0 (0) |
| Number of social workers |  |  |  |  |  |  |  |  |  |
| ≤1 | 9 (47) |  | 3 (75) |  | 1 (20) |  | 3 (50) |  | 2 (50) |
| >1 | 10 (53) |  | 1 (25) |  | 4 (80) |  | 3 (50) |  | 2 (50) |
|  |  |  |  |  |  |  |  |  |  |
| ***Activities*** |  |  |  |  |  |  |  |  |  |
| Number of ART-treated patients | |  |  |  |  |  |  |  |  |
| ≤1803 | 10 (53) |  | 0 (0) |  | 0 (0) |  | 6 (100) |  | 4 (100) |
| >1803 | 9 (47) |  | 4 (100) |  | 5 (100) |  | 0 (0) |  | 0 (0) |
| Subdivided biological check-up | |  |  |  |  |  |  |  |  |
| No | 13 (68) |  | 4 (100) |  | 3 (60) |  | 5 (83) |  | 1 (25) |
| Yes | 6 (32) |  | 0 (0) |  | 2 (40) |  | 1 (17) |  | 3 (75) |
| Educational support |  |  |  |  |  |  |  |  |  |
| No | 11 (58) |  | 1 (25) |  | 2 (40) |  | 6 (100) |  | 2 (50) |
| Yes | 8 (42) |  | 3 (75) |  | 3 (60) |  | 0 (0) |  | 2 (50) |
| Nutritional support |  |  |  |  |  |  |  |  |  |
| No | 6 (32) |  | 3 (75) |  | 0 (0) |  | 3 (50) |  | 0 (0) |
| Yes | 13 (68) |  | 1 (25) |  | 5 (100) |  | 3 (50) |  | 4 (100) |
| Financial support |  |  |  |  |  |  |  |  |  |
| No | 13 (68) |  | 3 (75) |  | 4 (80) |  | 2 (33) |  | 4 (100) |
| Yes | 6 (32) |  | 1 (25) |  | 1 (20) |  | 4 (67) |  | 0 (0) |
|  |  |  |  |  |  |  |  |  |  |
| ***Organization*** |  |  |  |  |  |  |  |  |  |
| Task-shifting for consultation and medical follow-up | | | |  |  |  |  |  |  |
| No | 7 (37) |  | 1 (25) |  | 2 (40) |  | 4 (67) |  | 0 (0) |
| Yes | 12 (63) |  | 3 (75) |  | 3 (60) |  | 2 (33) |  | 4 (100) |
| Task-shifting for prescription |  |  |  |  |  |  |  |  |  |
| No | 8 (42) |  | 3 (75) |  | 2 (40) |  | 3 (50) |  | 0 (0) |
| Yes | 11 (58) |  | 1 (25) |  | 3 (60) |  | 3 (50) |  | 4 (100) |
| Community-based organization involvement | |  |  |  |  |  |  |  |  |
| No | 9 (47) |  | 4 (100) |  | 0 (0) |  | 4 (67) |  | 1 (25) |
| Yes | 10 (53) |  | 0 (0) |  | 5 (100) |  | 2 (33) |  | 3 (75) |
| Specific ARV management |  |  |  |  |  |  |  |  |  |
| No | 5 (26) |  | 1 (25) |  | 0 (0) |  | 2 (33) |  | 2 (50) |
| Yes | 14 (74) |  | 3 (75) |  | 5 (100) |  | 4 (67) |  | 2 (50) |
|  |  |  |  |  |  |  |  |  |  |
| ***Technical capacities*** |  |  |  |  |  |  |  |  |  |
| Medical imaging equipment |  |  |  |  |  |  |  |  |  |
| No | 14 (74) |  | 4 (100) |  | 1 (20) |  | 5 (83) |  | 4 (100) |
| Yes | 5 (26) |  | 0 (0) |  | 4 (80) |  | 1 (17) |  | 0 (0) |
| CD4 count machine | |  |  |  |  |  |  |  |  |
| No | 10 (53) |  | 2 (50) |  | 2 (40) |  | 4 (67) |  | 2 (50) |
| Yes | 9 (47) |  | 2 (50) |  | 3 (60) |  | 2 (33) |  | 2 (50) |
| ARV stock-out |  |  |  |  |  |  |  |  |  |
| No | 9 (47) |  | 3 (75) |  | 3 (60) |  | 1 (17) |  | 2 (50) |
| Yes | 10 (53) |  | 1 (25) |  | 2 (40) |  | 5 (83) |  | 2 (50) |
| *Abbreviations: ARV = Antiretroviral drugs HSP: HIV-service profile.* | | | | | | | | | |
