# Supplementary material for: Individual and healthcare supply-related HIV transmission factors in HIV-positive patients enrolled in the antiretroviral treatment access program in the Centre and Littoral regions in Cameroon (ANRS-12288 EVOLCam survey)
Source: PLoS One. 2022 Apr 6;17(4):e0266451. doi: 10.1371/journal.pone.0266451 (PMC8985982; doi:10.1371/journal.pone.0266451)
Supplement: S2 Table — Abbreviation: NC: not concerned; MD: missing data; ART: antiretroviral treatment; HSP: HIV-service profile. (DOCX) [file pone.0266451.s002.docx]

**S2 Table. Description of the variables of interest for the description of the HIV transmission risk outcome (n=1372, EVOLCam survey, ANRS-12288)**

|  | **Total**  **N=1372** | **HIV transmission risk** | |
| --- | --- | --- | --- |
|  |  |  |  |
|  |  | **Yes**  **N=540 (39%)** | **No**  **N= 832 (61%)** |
|  | **n (%) or median [IQR]** | **n (%) or**  **median [IQR]** | **n (%) or**  **median [IQR]** |
| ***Demographic and socioeconomic characteris****tics* |  |  |  |
| Age (in years) | 39 [33-44] | 38 [32-43] | 39 [34-45] |
| Gender |  |  |  |
| Men | 447 (32.6%) | 138 (25.6%) | 309 (37.1%) |
| Women | 925 (67.4%) | 402 (74.4%) | 523 (62.9%) |
| Residential setting (MD=13) |  |  |  |
| Urban | 1154 (84.9%) | 446 (83.7%) | 708 (85.7%) |
| Rural | 205 (15.1%) | 87 (16.3%) | 118 (14.3%) |
| Marital status (MD=23) |  |  |  |
| Single | 193 (14.3%) | 79 (14.9%) | 114 (13.9%) |
| Married (legal or customary) | 539 (40.0%) | 178 (33.5%) | 361 (44.1%) |
| Common-law union | 617 (45.7%) | 274 (51.6%) | 343 (41.9%) |
| Having a main partner |  |  |  |
| Yes | 1179 (85.9%) | 461 (85.4%) | 718 (86.3%) |
| No | 193 (14.1%) | 79 (14.6%) | 114 (13.7%) |
| Living with main partner (MD=19) |  |  |  |
| Yes | 757 (55.9%) | 272 (51.0%) | 485 (59.1%) |
| No | 403 (29.8%) | 182 (34.1%) | 221 (27.0%) |
| Did not have a main partner | 193 (14.3%) | 79 (14.8%) | 114 (13.9%) |
| Number of children |  |  |  |
| None | 153 (11.2%) | 57 (10.6%) | 96 (11.5%) |
| 1-4 | 950 (69.2%) | 374 (69.3%) | 576 (69.2%) |
| ≥5 | 269 (19.6%) | 109 (20.2%) | 160 (19.2%) |
| Currently desiring or trying to have a child (MD=6) |  |  |  |
| Yes | 780 (57.1%) | 319 (59.4%) | 461 (55.6%) |
| No | 586 (42.9%) | 218 (40.6%) | 368 (44.4%) |
| Educational level (MD=7) |  |  |  |
| Lower than high school | 1225 (89.7%) | 488 (90.9%) | 737 (89.0%) |
| High school and above | 140 (10.3%) | 49 (9.1%) | 91 (11.0%) |
| Household monthly income per adult-equivalent (in USD) | 14.7 (21.8) | 14.9 (20.2) | 14.6 (6.9, 29.6) |
| Economic activity |  |  |  |
| Yes | 933 (68.0%) | 341 (63.1%) | 592 (71.2%) |
| No | 439 (32.0%) | 199 (36.9%) | 240 (28.8%) |
|  |  |  |  |

| ***Sexual behaviors and psychosocial characteristics*** |  |  |  |
| --- | --- | --- | --- |
| Number of sexual partners (12 previous months) |  |  |  |
| <2 | 1097 (80.0%) | 396 (73.3%) | 701 (84.3%) |
| ≥2 | 275 (20.1%) | 144 (26.7%) | 131 (15.8%) |
| Number of sexual partners in lifetime (MD=1) |  |  |  |
| ≤10 sexual partners | 959 (69.9%) | 382 (70.7%) | 577 (69.4%) |
| >10 male sexual partners | 108 (7.9%) | 49 (9.1%) | 59 (7.1%) |
| >10 female sexual partners | 202 (14.7%) | 76 (14.1%) | 126 (15.2%) |
| Unknown | 102 (7.4%) | 33 (6.1%) | 69 (8.3%) |
| Inconsistent condom use with the two previous sexual partners (MD=4) |  |  |  |
| No | 491 (35.8%) | 0 (0.0%) | 491 (59.2%) |
| Yes | 879 (64.2%) | 540 (100.0%) | 339 (40.8%) |
| HIV-status of the two previous sexual partners |  |  |  |
| Only HIV-positive | 343 (25.0%) | 0 (0.0%) | 343 (41.2%) |
| At least one negative or unknown HIV status sexual partner | 1029 (75.0%) | 540 (100.0%) | 489 (58.8%) |
| Inconsistent condom use with at least one negative or unknown HIV status sexual partner (MD=2) |  |  |  |
| No | 741 (54.1%) | 0 (0.0%) | 741 (89.3%) |
| Yes | 629 (45.9%) | 540 (100.0%) | 89 (10.7%) |
| Transactional sex (12 months prior to survey) |  |  |  |
| Yes (bought or sold sex) | 33 (2.4%) | 17 (3.2%) | 16 (1.9%) |
| No | 1334 (97.6%) | 521 (96.8%) | 813 (98.1%) |
| Mental quality of life (range 0-100) (per unit) | 45.0 (14.0) | 45.0 (14.0) | 45.0 (39.0, 52.0) |
| HIV-related stigma score (range 0-8) | 0.0 [0-1] | 0.0 [0-1] | 0.0 [0-0] |
| Binge drinking (MD=5) |  |  |  |
| < once a month | 1266 (92.6%) | 500 (93.1%) | 766 (92.3%) |
| At least once a month | 101 (7.4%) | 37 (6.9%) | 64 (7.7%) |
|  |  |  |  |
| ***Clinical characteristics*** |  |  |  |
| Time since HIV diagnosis (years) | 4.0 [2-7] | 3.0 [1-7] | 4.0 [2-7] |
| Disease symptoms at HIV diagnosis (MD=2) |  |  |  |
| No | 593 (43.3%) | 213 (39.5%) | 380 (45.7%) |
| Yes | 777 (56.7%) | 326 (60.5%) | 451 (54.3%) |
| Time between diagnosis and ART initiation (MD=3) |  |  |  |
| <2 months | 492 (35.9%) | 175 (32.5%) | 317 (38.2%) |
| ≥2 months | 753 (55%) | 296 (54.9%) | 457 (55.1%) |
| NC (not treated) | 124 (9.1%) | 68 (12.6%) | 56 (6.7%) |
| CD4 count at ART initiation (MD=105) |  |  |  |
| ≥100 cells/mm3 | 866 (68.4%) | 331 (65.3%) | 535 (70.4%) |
| <100 cells/mm3 | 277 (21.9%) | 108 (21.3%) | 169 (22.2%) |
| NC (not treated) | 124 (9.8%) | 68 (13.4%) | 56 (7.4%) |
| On ART for at least 6 months (MD=6) |  |  |  |
| Yes | 1119 (81.7%) | 403 (74.8%) | 716 (86.3%) |
| On ART for <6 months | 126 (9.2%) | 68 (12.6%) | 58 (7.0%) |
| Not on ART | 124 (9.1%) | 68 (12.6%) | 56 (6.8%) |
| Adherence to treatment in patients on ART for at least 6 months (MD=6) |  |  |  |
| Low | 823 (60.1%) | 376 (69.8%) | 447 (53.9%) |
| High | 296 (21.6%) | 27 (5.0%) | 269 (32.4%) |
| Not on ART ≥6 months | 250 (18.3%) | 136 (25.2%) | 114 (13.7%) |
| HIV viral load in patients on ART for at least 6 months (MD=24) |  |  |  |
| Undetectable | 796 (58.5%) | 249 (46.4%) | 547 (66.5%) |
| Detectable | 314 (23.1%) | 152 (28.3%) | 162 (19.7%) |
| NC (not on ART for ≥6 months) | 250 (18.4%) | 136 (25.3%) | 114 (13.9%) |
| Aviremic status (MD=2) |  |  |  |
| Stable | 219 (16.0%) | 0 (0.0%) | 219 (26.4%) |
| Unstable | 1152 (84.0%) | 540 (100.0%) | 612 (73.6%) |
|  |  |  |  |
| ***Healthcare service characteristics*** |  |  |  |
| HIV-service profiles |  |  |  |
| HSP1 (n=4) | 222 (16.2%) | 72 (13.3%) | 150 (18.0%) |
| HSP2 (n=5) | 414 (30.2%) | 167 (30.9%) | 247 (29.7%) |
| HSP3 (n=6) | 451 (32.9%) | 192 (35.6%) | 259 (31.1%) |
| HSP4 (n=4*)* | 285 (20.8%) | 109 (20.2%) | 176 (21.2%) |
| *Abbreviation: NC: not concerned; MD: missing data; ART: antiretroviral treatment; HSP: HIV-service profile.* | | | |
