# Supplementary material for: Individual and healthcare supply-related HIV transmission factors in HIV-positive patients enrolled in the antiretroviral treatment access program in the Centre and Littoral regions in Cameroon (ANRS-12288 EVOLCam survey)
Source: PLoS One. 2022 Apr 6;17(4):e0266451. doi: 10.1371/journal.pone.0266451 (PMC8985982; doi:10.1371/journal.pone.0266451)
Supplement: S1 Fig — (DOCX) [file pone.0266451.s003.docx]

**S1 Fig. Multiple correspondence analysis (MCA) coordinate plots of categories of active variables (characteristics of centers) (EVOLCam survey, ANRS 12288)**
