# Supplementary material for: Individual and healthcare supply-related HIV transmission factors in HIV-positive patients enrolled in the antiretroviral treatment access program in the Centre and Littoral regions in Cameroon (ANRS-12288 EVOLCam survey)
Source: PLoS One. 2022 Apr 6;17(4):e0266451. doi: 10.1371/journal.pone.0266451 (PMC8985982; doi:10.1371/journal.pone.0266451)
Supplement: S2 Fig — (DOCX) [file pone.0266451.s004.docx]

**S2 Fig. Multiple correspondence analysis (MCA) coordinate plots of centers (as supplementary variable) and clustering: HSP 1 (S1, S2, S3, S4), HSP 2 (S5, S6, S7, S8, S9), HSP 3 (S10, S11, S12, S13, S14, S15), HSP 4 (S16, S17, S18, S19) (EVOLCam survey, ANRS 12288).**
